# Epidemiology of nontuberculous mycobacteria among patients with cystic fibrosis in Scandinavia

**DOI:** 10.1016/j.jcf.2014.08.002

**Published:** 2015-01

**Authors:** Tavs Qvist, Marita Gilljam, Bodil Jönsson, David Taylor-Robinson, Søren Jensen-Fangel, Mikala Wang, Anita Svahn, Karsten Kötz, Lennart Hansson, Annika Hollsing, Christine R. Hansen, Pål L. Finstad, Tania Pressler, Niels Høiby, Terese L. Katzenstein, on behalf of the Scandinavian Cystic Fibrosis Study Consortium (SCFSC)

**Affiliations:** a Copenhagen CF Center, Department of Infectious Diseases, University Hospital Rigshospitalet, Denmark; b Gothenburg CF Center, Department of Respiratory Medicine, Sahlgrenska University Hospital, Gothenburg, Sweden; c Clinical Microbiology Laboratories, Sahlgrenska University Hospital, Gothenburg, Sweden; d Department of Public Health and Policy, University of Liverpool, Liverpool, UK; e Department of Infectious Diseases, Aarhus University Hospital, Denmark; f Department of Clinical Microbiology, Aarhus University Hospital, Denmark; g Department of Clinical Microbiology, Karolinska University Hospital, Solna, Sweden; h Gothenburg CF Center, Department of Pediatrics, Sahlgrenska University Hospital, Gothenburg, Sweden; i Lund CF Center, Department of Respiratory Medicine and Allergology, Skane University Hospital, Sweden; j Uppsala CF center, KBH, Uppsala University Hospital, Sweden; k Department of Pediatrics, Copenhagen University Hospital Rigshospitalet, Denmark; l Norwegian Resource Centre for Cystic Fibrosis, Oslo University Hospital, Oslo, Norway; m Department of Clinical Microbiology, Copenhagen University Hospital Rigshospitalet, Denmark

**Keywords:** *Mycobacterium abscessus*, *Mycobacterium avium*, Prevalence, Susceptibility

## Abstract

**Background:**

Nontuberculous mycobacteria (NTM) are an emerging threat to cystic fibrosis (CF) patients but their epidemiology is not well described.

**Methods:**

In this retrospective observational study we identified all Scandinavian CF patients with a positive NTM culture from airway secretions from 2000 to the end of 2012 and used national CF databases to describe microbiological and clinical characteristics.

**Results:**

During the 13-year period 157 (11%) CF patients were culture positive for NTM at least once. *Mycobacterium abscessus* complex (MABSC) (45%) and *Mycobacterium avium* complex (MAC) (32%) were the predominant species with geographical differences in distribution. Younger patients were more prone to MABSC (*p* < 0.01). Despite treatment, less than one-third of MABSC patients with repeated positive cultures cleared their infection and a quarter had a lung transplant or died.

**Conclusion:**

NTM are significant CF pathogens and are becoming more prevalent in Scandinavia. MABSC and MAC appear to target distinct patient groups. Having multiple positive cultures despite treatment conveys a poor outcome.

## 1 Introduction

The clinical significance of a nontuberculous mycobacteria (NTM) positive culture from airway secretions of cystic fibrosis (CF) patients remains uncertain [1]. For some, a positive NTM culture coincides with clinical deterioration, while for others, culture positivity seems to be transient and of no clinical importance [1]. If little is known about the significance of a positive culture [2], even less is known about patient susceptibility and risk factors for acquisition [3]. Understanding the risks associated with infection is particularly important due to the well-described adverse effects of current NTM treatment regimens [4,5]. In light of the recent attention NTM is receiving as a threat to CF patients [6], we examined the situation in Scandinavia. The objective of this retrospective multicenter study was to describe the scope and importance of NTM in the complete Scandinavian CF population. We thus report, for the first time, the prevalence of NTM among all CF patients in Scandinavia; geographical differences in species distribution; explore risk factors for acquisition; and describe outcomes including development of end-stage-lung-disease (ESLD).

## 2 Methods

### 2.1 Setting

Scandinavia consists of Denmark, Norway and Sweden and has a total population of 20.2 million people [7], with an estimated CF incidence of 1 in 4900 live births [8,9] and generally a high proportion of patients who are homozygous for the F508del mutation in the CF transmembrane conductance regulator (CFTR) gene (87%, 71%, 67% for Denmark, Norway, Sweden) [10]. All CF patients receive treatment at one of eight CF centers, where patients are seen on an outpatient basis on average eight times annually. Patients with less than 8 scheduled annual visits (Norway, Sweden), were seen additionally at local hospitals as part of shared care agreements, implemented to reduce long distance travel. With the exception of Denmark, patients were screened for NTM annually either by expectorated sputum, laryngeal suction or bronchoalveolar lavage (BAL), starting at age 6 to 10 years of age with some variability in screening policies over time and between centers. In Denmark, systematic annual screening was introduced in 2011; prior to this, patients were only cultured for NTM upon clinical suspicion. During visits, anthropometric data and forced expiratory volume in 1 s (FEV1) were recorded. Pulmonary function tests were performed according to international recommendations [11], measuring FEV1, expressed as a percentage of predicted values for sex and height, using reference equations from Wang or Hankinson [12,13].

### 2.2 NTM isolation

Depending on CF center, sputum and BAL samples were pretreated with SDS-NaOH, a cocktail of antibiotics (amphotericin B, carbenicillin, polymyxin B-sulfate and trimethoprim-lactate), or 5% oxalic acid. Samples were then cultured for mycobacteria on either Löwenstein–Jensen egg medium, Bactec 460™ culture system, MGIT™ (Mycobacteria Growth Indicator Tube) 960 system or *Burkholderia cepacia* selective agar [14]. In most centers, more than one method of decontamination and culture was used reflecting ongoing advances in methodology. Positive cultures were identified to species-level by 16-23S spacer array technique, sequencing of the *rpoB* gene, hybridization and/or growth ability on Löwenstein–Jensen slants with 5% NaCl. In some centers, the GenoType Mycobacterium CM and AS tests [15] and DNA sequencing of the *hsp65* gene [16] were used.

### 2.3 Prevalence study

In the first part of the study we aimed to describe the prevalence as well as geographical and species distribution of NTM in Scandinavia. CF registries and microbiological databases in Denmark, Norway and Sweden were queried for any patient with CF, who had at least one positive NTM airway culture from January 2000 to December 2012. The American Thoracic Society (ATS) and Infectious Disease Society of America (IDSA)'s microbiological criteria were used to classify patients [17], dividing NTM patients into those with only a single positive sputum culture, and those with > 1 positive sputum culture or at least one positive BAL.

### 2.4 Comparison with background Scandinavian CF population

In the second part of the study we aimed to describe the clinical characteristics of patients at time of first NTM positive culture. For comparison purposes, similar data from a recent clinical study of the Scandinavian CF population were captured [10], including sex, age, and CFTR mutation data. Prior Gram-negative infection, pancreatic insufficiency (PI), azithromycin and steroid maintenance treatment as well as CF-related diabetes mellitus (CFRD) diagnosed using previously published criteria [18], were all also included at time of first positive NTM culture for cases, and in the calendar year 2002 for the background population. We stratified the analysis according to NTM species to examine whether different NTM species were more common in CF patients with particular clinical characteristics.

### 2.5 Outcome study

The third part of the study aimed to describe the clinical impact of NTM disease. We therefore examined number of positive cultures, NTM treatment (yes, no), duration of infection, development of end stage lung disease (ESLD) and clearance of NTM, stratified by age group (< 20 years vs. ≥ 20 years of age), gender, NTM species and ATS/IDSA criteria. ESLD was defined as lung transplantation or death, whichever came first. To distinguish patients where NTM involvement was judged to have played a role in the development of ESLD, data was further subdivided depending on clinical certainty of NTM involvement in death or lung transplantation. Time of NTM clearance was defined as the first calendar year without culture positivity, if the patient was off treatment.

### 2.6 Statistical methods

Baseline data were calculated as medians and interquartile ranges (IQR) for continuous variables, and percentages for categorical variables. Cross sectional group comparisons were made using analysis of variance or Kruskal Wallis non-parametric tests. Differences in characteristics between groups were evaluated using the Chi-square test and binary logistic regression was used to adjust for statistically significant covariates in the outcome analysis. A level of 0.05 was set for statistical significance. All statistical analyses were performed using SPSS version 19.0 (SPSS Inc., Chicago, IL).

### 2.7 Ethical considerations

Ethical approval was not required for the Scandinavian data collection. Data was housed in Copenhagen, Denmark as approved by the Danish Data Protection Agency (File: 2007-58-0015).

## 3 Results

### 3.1 Prevalence study

**Fig. 1:**
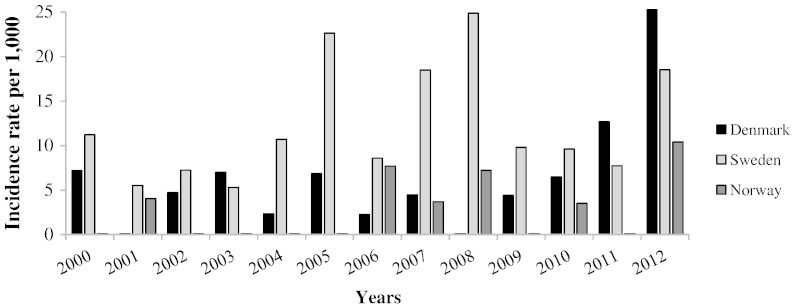
New cases of patients with at least one nontuberculous mycobacteria positive culture among CF patients in Scandinavia.

**Fig. 2:**
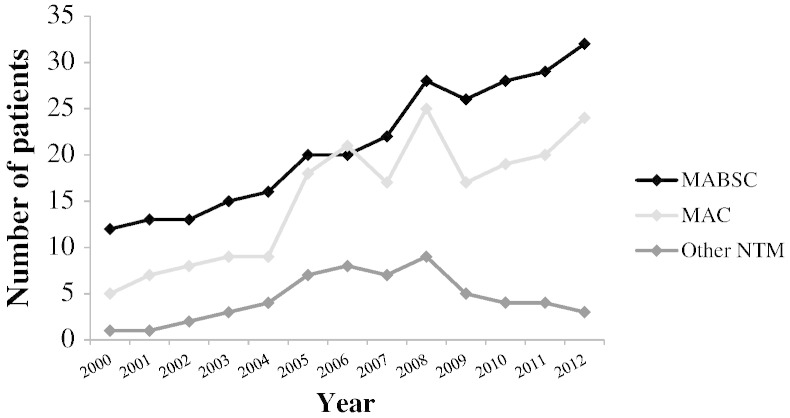
Number of CF patients fulfilling ATS/IDSA microbiological criteria for nontuberculous mycobacteria infection in Scandinavia from 2000 to 2012.

From 2000 to the end of 2012, Scandinavia's CF population increased from 1197 to 1411 individuals. During this time, 14,385 NTM cultures were performed on 1270 patients giving a mean of 11 (range: 1–60) cultures per patient during the 13-year period. NTM was cultured 1193 times from 157 subjects giving an overall period prevalence of patients with at least one positive NTM culture of 11% (157/1411). The annual number of new patients with NTM increased during the study period with a mean of 8 new cases annually from 2000–2006 to a mean of 14.5 new cases annually between 2007 and 2012. However the CF population in Scandinavia also grew 15% during the period. Fig. 1 shows the number of new cases adjusted for annual population size (NTM incidence) and stratified by country. Norway and Sweden thus did not see a consistently rising incidence over the period, but rather a fluctuating number of new cases with notable peaks in 2005, 2007 and 2008. Denmark saw a sharp increase in incidence in the last two years of the period, but this coincided with the implementation of intensified screening procedures, going from only testing patients suspected of NTM infection, to introducing annual screening of all patients. Norway and Sweden performed annual screening throughout the study period. In 7 out of 8 CF centers, pediatric patients had sputum screened starting at 6–10 years of age. One center (Copenhagen) used laryngeal suction starting from infancy. The annual prevalence rate of *Mycobacterium abscessus complex* (MABSC) and *Mycobacterium avium complex* (MAC) increased in all three countries as patients accumulated throughout the study period. Thus in 2000 1.7% of the Scandinavian CF population fulfilled the ATS/IDSA microbiological criteria, a figure which grew to 4.1% by the end of 2012. Fig. 2 illustrates the steady rise in the number of NTM patients each year of the study period. NTM were recovered from subjects in all participating centers, but period prevalence rates varied by geographic location, ranging from 3% in Aarhus, Denmark to 28% in Gothenburg, Sweden. Likewise the distribution of species varied among sites, with MABSC being the predominant NTM in Denmark and MAC being more dominant in Norway and in Sweden, which also had a higher rate of patients with two different NTM species, typically a combination of MABSC and MAC (Table 1). Overall, 70 patients grew only MABSC making it the most common NTM species (45%), followed by MAC, which was cultured exclusively from 51 patients (32%). An additional 17 patients had both MABSC and MAC (11%). Ten patients had NTM species other than MASBC and MAC including *Mycobacterium lentiflavum*, *Mycobacterium malmoense*, *Mycobacterium fortuitum*, *Mycobacterium kansasii* or unknown mycobacterial species. The remaining nine patients had combinations of the NTM species mentioned above or combinations with isolates of *Mycobacterium triplex*, *Mycobacterium nebraskense* and *Mycobacterium gordonae*. Subspeciation of MABSC was not introduced in Scandinavia until after 2012 and subspecies of MAC could not be reliably compared due to historical differences in taxonomy between centers. Some MABSC cultures from the early period were classified as *Mycobacterium chelonae*, but were later reclassified as MABSC in accordance with changes in mycobacterial taxonomy [19].

The median age of patients at time of first positive NTM culture was 19 years (IQR: 13–29 years) and 55% were male. The median %FEV1 was 82 (IQR: 61–97), and median Z-BMI was − 0.4 (IQR: − 0.9–0.5). Seventy-one patients (45%) had a concurrent chronic bacterial infection, most frequently with *Pseudomonas aeruginosa* (34%). Seventy of the 157 patients had data available on azithromycin use. Of these, 19 patients (27%) were in continuous azithromycin treatment at the time of first positive NTM culture.

### 3.2 Comparison with background Scandinavian CF population

The characteristics of the 157 NTM patients were compared with a previous cross sectional study of 989 patients, representing 86% of the CF population in Scandinavia in 2002 [10]. NTM cases were more likely than the background CF population to have diabetes (23% vs. 11%, *p* < 0.001), but were not distinguishable in other respects. This finding remained significant after adjustment for age and when including only patients who fulfilled ATS/IDSA microbiological criteria (*p* < 0.01) (Table 2).

**Fig. 3:**
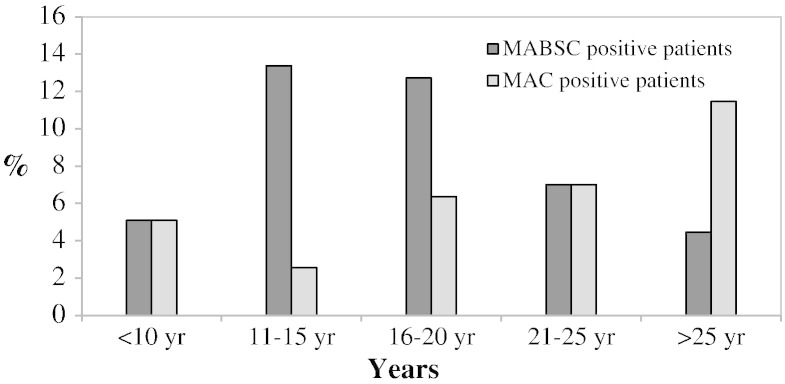
Distribution of *Mycobacterium abscessus* complex (MABSC) and *Mycobacterium avium* complex (MAC) positive patients according to age at time of first isolate.

To compare the characteristics of patients with MABSC and MAC, patients with other NTM species (n = 10) and co-infected patients (n = 26) were excluded (Table 3). MABSC was acquired at an earlier age than MAC (median age 17 vs. 22 years, *p* < 0.01) (Fig. 3) and there was a trend towards MABSC patients being more likely to have pancreatic insufficiency (94% vs. 84%, *p* = 0.07). No other significant differences in clinical characteristics could be identified (Tables 2 and 3).

### 3.3 Outcome study

Of the 157 NTM patients, 125 (80%) fulfilled ATS/IDSA microbiological criteria during the study period. These patients were NTM positive for a total of 472 person-years, of which the majority was contributed by 69 patients, who had ≥ 5 positive NTM cultures (Table 3).

The median duration of infection was 2 years (IQR: 1–6 years) for all 125 patients fulfilling ATS/IDSA microbiological criteria and 5 years (IQR: 2–9) for the group with ≥ 5 positive cultures (*p* < 0.001). NTM acquisition before the age of 20 (NTM < 20 years) was more common in MABSC patients compared to MAC (88% vs. 38% *p* < 0.001) and in patients who went to fulfill ATS/IDSA microbiological criteria (85% vs. 71%, *p* = 0.04) with a median of 5 positive NTM cultures (IQR: 2–13) compared to a median of 3 positive cultures (IQR: 1–6) (*p* = 0.01) in the group who acquired NTM as adults. However, the association between NTM < 20 years and fulfilling ATS/IDSA criteria was no longer significant, when patients were stratified for NTM species. No gender differences were observed.

Out of the 107 patients (86%) for whom treatment data were available, 79 (74%) received some form of NTM treatment. Forty-nine patients (39%) had a successful outcome i.e. became culture negative, either following treatment (n = 24/125, 19%), or without treatment (n = 18/125, 14%). Of the 58 patients (46% of those fulfilling ATS criteria) who did not clear their NTM by the end of the study period, 46 (79%) had received NTM treatment. Overall more patients with MABSC than with MAC received treatment for their infection (72% vs. 44%, *p* = 0.02). A higher proportion of those who acquired NTM before the age of 20 ended up developing ESLD (19% vs. 13%), but this was not a statistically significant difference (*p* = 0.32). MABSC patients with a history of more than 4 positive cultures were most likely to have received treatment (93%), least likely to clear infection (16%) and had the highest rate of ESLD (26%), although these findings did not reach statistical significance (Table 4).

## 4 Discussion

In the Scandinavian CF population MABSC appears to be more common in younger patients. By contrast MAC is generally acquired later in life and possibly by patients with a less severe form of CF as indicated by a trend towards a higher proportion of pancreatic sufficiency in this group. This corroborates a previous study from France [20]. Furthermore we suggest a role of diabetes as a risk factor for NTM acquisition; a finding that warrants further exploration. MABSC patients with repeated positive cultures had the worst outcomes with one-forth progressing to ESLD.

### 4.1 Prevalence

In Scandinavia, reported incidence rates of MABSC increased in the two CF centers that introduced intensified screening, while fluctuations in incidence were seen in six other centers with considerable geographical and species variation. Still, due to an accumulation effect, the total number of patients with NTM increased, so that by the end of the study period 11% of the Scandinavian CF population had been culture positive at least once. In comparison, two smaller prospective studies of NTM from 1990 (Sweden) [21] and 1993 (Denmark) [22], found NTM prevalence rates of 9% and 4%, respectively. Since 1980, 19 studies from primarily the US and Europe have reported prevalence rates of NTM among CF patients [3], with large geographical and inter-center variation (median 9% IQR: 6% − 14%). Likewise, in Scandinavia we see considerable geographical variation in the prevalence of NTM (range per center: 3%–28%). MABSC was the predominant NTM species in Denmark, whereas MAC was slightly more prevalent in Norway and Sweden. While environmental factors are often mentioned as a possible explanation for differences in species distribution [23], differences in culture frequency and technique between centers may explain the differences observed. For example one center which only reported MABSC cases, primarily used a culture technique suitable for fast growing mycobacteria [14]; an approach likely to miss MAC. Previously, automated liquid culture platforms have been reported to cause reduced recovery of NTM and produce a high number of contaminated cultures [24], particularly from CF patients [25].

### 4.2 Risk of NTM acquisition

MABSC patients were younger than MAC patients and we observed a trend towards a higher likelihood of pancreatic insufficiency among MABSC patients. This corroborates the findings of a French nested case–control by Catherinot et al. from 2013 [20]. The reasons for these differences remain unknown. To further explore association between clinical characteristics and NTM acquisition we compared NTM cases to the background CF population. Despite a suboptimal design, where NTM patients comprised both the case and a small part of the control group, the analysis suggested that NTM patients in Scandinavia were more likely to have diabetes than the background CF population despite a similar median age. This link has been shown previously in a smaller CF patient sample [26] and warrants further exploration, ideally in a prospective design. The fact that no other statistically significant associations were observed should be interpreted with caution, as the design tended to underestimate any association. Likewise, any risk-inferring or protective role of azithromycin maintenance therapy at time of first positive culture could not be determined. The role azithromycin exposure may play in causing NTM disease remains controversial [27,28]. Azithromycin policies varied between the centers during the study period. In four centers, azithromycin was given to all CF patients with chronic *P. aeruginosa* lung infection and the remaining centers had a variable azithromycin policy as previously described [10].

### 4.3 Outcomes

MABSC patients had a higher number of positive cultures and were more likely to receive treatment, a finding in line with the general consensus that MABSC infection is associated with clinical deterioration [26]. Likewise MABSC patients with a history of more than 4 positive cultures were the least likely to clear infection and had the highest rate of ESLD.

Study limitations include the retrospective design and lack of data on smear positivity and colony morphology, factors which have been identified as important in previous studies [29–32]. Furthermore our outcome analysis did not include important factors such as lung function data; subsequent acquisition of other infections; the specific nature of NTM treatment regimens and additionally ATS/IDSA radiological criteria could not be applied; all due to limitations in study design. However, Scandinavia provides an ideal setting for CF studies as treatment is restricted to eight medical centers and all CF patients are seen with an unparalleled frequency. The primary strength of this study is thus the inclusion of three complete national CF populations in a comprehensive 13-year study period.

### 4.4 Conclusion

This study confirms that NTM are important CF pathogens in Scandinavia with 11% of patients having been culture positive at least once between 2000 and the end of 2012. NTM species exhibit geographical differences and MABSC and MAC appear to target distinct CF patient subpopulations, with younger, pancreatic insufficient patients being more prone to MABSC culture positivity. Despite treatment, less than a third of MABSC patients with repeated positive cultures cleared their infection and one-fourth were eventually lung transplanted or died.

## Conflict of interest

None of the authors report conflicts of interest.

## Sources of support

DTR is supported by an MRC Population Health Scientist Fellowship (G0802448).

## Author contributions

**Table:** 

Idea and conceptualization:	Qvist T, Pressler T, Jönsson B, Gilljam M, Katzenstein TL
Microbiological data collection:	Svahn A, Wang M, Jönsson B, Høiby N, Qvist T
Clinical data collection:	Kötz K, Hansson L, Hollsing A, Finstad PL, Laerum BL, L Hansson, Jönsson B, Gilljam M, Jensen-Fangel S, Katzenstein TL, Qvist T
Data analysis:	Qvist T, Hansen CR, Taylor-Robinson D
All authors participated in revising the manuscript and have approved the final version.	

## Figures and Tables

**Table 1:** Species and geographical distribution of nontuberculous mycobacteria in Scandinavia.

Country	CF Center		NTM prevalence 2000–2012	MABSC (% a)	No. of patients with ≥ 1		Positive culture
					MAC	Other NTM species b	More than one NTM species c
Denmark	Aarhus		3%	4 (80)	0	0	1
	Copenhagen		13%	29 (69)	12	0	1
Norway	Bergen		5%	1 (50)	1	0	0
	Oslo		4%	3 (30)	5	1	1
Sweden	Uppsala		5%	2 (40)	1	1	1
	Stockholm		13%	10 (34)	7	4	8
	Gothenburg		28%	15 (33)	20	0	10
	Lund		11%	5 (28)	5	4	4
	Total		11%	70 (45)	51	10	26

CF = cystic fibrosis, NTM = nontuberculous mycobacteria, MABSC = *M. abscessus* complex; MAC = *M. avium* complex.

a Patients exclusively infected with MABSC expressed as % of total NTM cases.

b *M. lentiflavum* (n = 2), *M. malmoense* (n = 2), *M. fortuitum* (n = 1), *M. kansasii* (n = 1) or unknown species (n = 4).

c 17/26 were combinations of exclusively MABSC and MAC.

**Table 2:** Characteristics of NTM cases at time of first positive culture compared to a cross section of the background Scandinavian CF population.

	NTM cases 2000–2012	CF population in 2002	*p*-Value
	*n* = 157	*n* = 989 a	
Median age, y (IQR)	19 (13–22)	17 (10–27)	0.23
Female, %	45	48	0.48
Homozygous for F508del mutation, %	61	53	0.10
Median FEV1% of predicted, (IQR)	82 (61–97)	80 (58–95)	0.44
Median Z-BMI (IQR)	− 0.4 (− 0.9–0.5)	− 0.2 (− 1.0–0.5)	0.43
Pancreatic insufficiency, n (%)	87	90	0.22
CFRD, %	23	11	< 0.01
Chronic Gram-negative infection, %	49	44	0.29
Continuous macrolides, %	24	24	0.88
Oral steroid treatment, %	7	4	0.11

a Captured from cross sectional study of 86% of the Scandinavian CF population in 2002, including NTM cases who could not be distinguished. CF = cystic fibrosis, BMI = body mass index, and CFRD = cystic fibrosis related diabetes.

**Table 3:** Characteristics of MABSC and MAC positive CF patients in Scandinavia.

Characteristic at time of first positive isolate	Patients with MABSC	Patients with MAC	*p*-Value
	(*n* = 70)	(*n* = 51)	
Female, %	52	38	0.13
Median age (IQR), y	17 (12–21)	22 (15–32)	< 0.01
Homozygous for F508del mutation, %	64	68	0.63
Median %FEV1 of pred. (IQR)	83 (57–96)	76 (62–95)	0.18
Median BMI kg/m^2^ (IQR)	19 (17–21)	20 (19–23)	0.11
Z-BMI	− 0.1 (− 1.0–0.6)	− 0.4 (− 1.0–0.5)	0.77
Pancreatic insufficiency, %	94	84	0.07
CFRD, %	22	18	0.62
Chronic Gram negative infection, %	59	69	0.33
*Aspergillus* infection a, %	48	55	0.13
ABPA, %	25	21	0.64
Azithromycin treatment, %	18	25	0.19
Oral or IV steroid treatment, %	10	7	0.63

MABSC = *M. abscessus* complex; MAC = *M. avium* complex; NTM = nontuberculous mycobacteria; IQR = interquartile range, CFRD = cystic fibrosis related diabetes.

a Defined as having positive *Aspergillus* cultures at least 6 months of a calendar year; ABPA = allergic bronchopulmonary aspergillosis, defined according to consensus criteria [33].

**Table 4:** Outcomes of 125 patients with more than 1 positive culture for nontuberculous mycobacteria from 2000 to 2012.

	MABSC		MAC		Other NTM/co-infected^a^	
	2–4 pos	≥ 5 pos	2–4 pos	≥ 5 pos	2–4 pos	≥ 5 pos
Number of patients	23	34	17	19	16	16
Median duration of infection, y	1	5	1	3	2	6
Received NTM treatment, n (%) b	13/19 (68%)	28/30 (93%)	5/14 (36%)	15/17 (88%)	4/11 (36%)	14/16 (88%)
Cleared infection, n (%) c	8/15 (53%)	5/31 (16%)	10/17 (59%)	8/19 (42%)	8/16 (50%)	8/16 (50%)
ESLD, all, n (%)	5 (22%)	9 (26%)	1 (6%)	4 (16%)	3 (19%)	2 (13%)
ESLD judged NTM related, n (%)	1 (4%)	8 (24%)	0	3 (16%)	1 (6%)	2 (13%)

a 32 patients who had NTM species other than MABSC and MAC or combinations of more than one NTM species.

b Data missing for 18 patients.

c 11 patients excluded due to too recent infection, MABSC = *M. abscessus* complex, MAC = *M. avium* complex, IQR = Interquartile range, ESLD = end stage lung disease, defined as death or lung transplantation during the study period.
